# Vaccinating humanitarian workers against COVID-19

**DOI:** 10.2471/BLT.23.289980

**Published:** 2023-11-21

**Authors:** Anne-Gaelle Selod, Jaclyn Perhati, Cedric Dumont, Baptiste Danjou, Daniel Cook

**Affiliations:** a Pan American Health Organization, 525 23rd Street NW, Washington, DC 20037, United States of America (USA).; b United Nations Medical Directors Group, United Nations, New York, USA.; c Department of Operational Support, United Nations Secretariat, New York, USA.

## Abstract

**Objective:**

To describe the United Nations’ (UN’s) coronavirus disease 2019 (COVID-19) vaccination programme and its efforts to vaccinate frontline humanitarian personnel stationed in locations where access to COVID-19 vaccine was limited or absent.

**Methods:**

The vaccination programme was structured as a two-level operation: a global vaccine deployment support team and local vaccine deployment teams in each participating country, territory or administrative area. The central group, led by a global vaccine coordinator, oversaw medical, legal, financial, logistical, data, technological and communication aspects. Local vaccine deployment teams were led by coordinators who managed registration, logistics, communication and vaccine administration. The programme used World Health Organization-approved COVID-19 vaccines and developed prioritization criteria for distributing vaccine supplies. The programme ensured that vaccines for the UN personnel were not diverted from the populations they were intended to serve.

**Findings:**

The programme successfully formed 120 deployment teams across 152 eligible countries, territories and administrative areas, targeting approximately 673 000 individuals. By April 2023, 72 countries, territories and administrative areas had received over 470 000 doses, of which 337 072 doses were administered. Almost half of the doses administered (167 616) were to individuals in five UN hardship countries. Ninety-five severe adverse events were reported, but none led to any reported medical evacuation, permanent disability or death.

**Conclusion:**

The programme demonstrated effective global coordination and local implementation, adapting to diverse contexts and operational challenges. The model can serve as a guide for global actors for future health emergencies, or for deploying health aid at a regional or global scale.

## Résumé

**Objectif:**

Décrire le programme de vaccination contre la maladie à coronavirus 2019 (COVID-19) de l’Organisation des Nations Unies (ONU) et ses efforts pour vacciner le personnel humanitaire de première ligne stationné dans des endroits où l’accès au vaccin contre la COVID-19 était limité ou inexistant.

**Méthodes:**

Le programme de vaccination était structuré comme une opération à deux niveaux : une équipe mondiale de soutien au déploiement des vaccins et des équipes locales de déploiement des vaccins dans chaque pays, territoire ou zone administrative participant. Le groupe central, dirigé par un coordinateur mondial des vaccins, supervisait les aspects médicaux, juridiques, financiers, logistiques, ainsi que ceux liés aux données, à la technologie et à la communication. Les équipes locales de déploiement des vaccins étaient dirigées par des coordinateurs chargés de gérer l’enregistrement, la logistique, la communication et l’administration des vaccins. Le programme a utilisé des vaccins COVID-19 approuvés par l’Organisation mondiale de la santé et a défini des critères de priorité pour la distribution des vaccins. Il veillait à ce que les vaccins destinés au personnel de l’ONU ne soient pas détournés des populations auxquelles ils étaient destinés.

**Résultats:**

Le programme a réussi à former 120 équipes de déploiement dans 152 pays, territoires et zones administratives admissibles, ciblant environ 673 000 individus. En avril 2023, 72 pays, territoires et zones administratives avaient reçu plus de 470 000 doses, dont 337 072 ont été administrées. Près de la moitié des doses administrées (167 616) l’ont été à des individus vivant dans cinq pays en difficulté tels qu’identifiés par l’ONU. Quatre-vingt-quinze effets indésirables graves ont été signalés, mais aucun n’aentraîné une évacuation médicale, une incapacité permanente ou le décès.

**Conclusion:**

Le programme a fait preuve d’efficacité dans la coordination mondiale et dans la mise en œuvre locale, en s’adaptant à divers contextes et défis opérationnels. Ce modèle peut servir de guide aux acteurs mondiaux pour les futures urgences sanitaires ou pour le déploiement d’une aide sanitaire à l’échelle régionale ou mondiale.

## Resumen

**Objetivo:**

Describir el programa de vacunación contra la enfermedad por coronavirus de 2019 (COVID-19) de las Naciones Unidas (ONU) y sus esfuerzos para vacunar al personal humanitario de primera línea ubicado en lugares donde el acceso a la vacuna contra la COVID-19 era limitado o inexistente.

**Métodos:**

El programa de vacunación se estructuró en una operación a dos niveles: un equipo de apoyo al despliegue mundial de vacunas y equipos locales de despliegue de vacunas en cada país, territorio o área administrativa participante. El grupo central, dirigido por un coordinador de vacunas a nivel mundial, supervisaba los aspectos médicos, jurídicos, financieros, logísticos, de datos, tecnológicos y de comunicación. Los equipos locales de despliegue de vacunas estaban dirigidos por coordinadores que gestionaban el registro, la logística, la comunicación y la administración de las vacunas. El programa utilizó vacunas contra la COVID-19 aprobadas por la Organización Mundial de la Salud y desarrolló criterios de priorización para distribuir los suministros de vacunas. El programa garantizó que las vacunas para el personal de la ONU no se desviaran de las poblaciones a las que estaban destinadas.

**Resultados:**

El programa formó con éxito 120 equipos de despliegue en 152 países, territorios y áreas administrativas elegibles, dirigidos a aproximadamente 673 000 personas. En abril de 2023, 72 países, territorios y áreas administrativas habían recibido más de 470 000 dosis, de las cuales 337 072 fueron administradas. Casi la mitad de las dosis administradas (167 616) correspondieron a personas de cinco países en situaciones apremiantes de las Naciones Unidas. Se notificaron 95 efectos adversos graves, pero ninguno dio lugar a evacuación médica, discapacidad permanente o muerte.

**Conclusión:**

El programa demostró que la coordinación mundial y la implementación local son eficaces, ya que se adapta a diversos contextos y desafíos operativos. El modelo puede servir de guía a los agentes de todo el mundo para futuras emergencias sanitarias o para el despliegue de ayuda sanitaria a escala regional o mundial.

## ملخص

**الغرض:**

وصف برنامج التطعيم ضد مرض فيروس كورونا 2019 (كوفيد 19) التابع للأمم المتحدة، وجهوده لتطعيم العاملين في المجال الإنساني في الخطوط الأمامية المتواجدين في المواقع التي تكون فيها إمكانية الحصول على لقاح كوفيد 19 محدودة أو معدومة.

**الطريقة:**

تم هيكلة برنامج التطعيم كعملية تتكون من مستويين: فريق عالمي لدعم توزيع اللقاح، وفرق محلية لتوزيع اللقاح في كل بلد أو إقليم أو منطقة إدارية مشاركة. وأشرفت المجموعة المركزية، بقيادة منسق عالمي للقاحات، على الجوانب الطبية، والقانونية، والمالية، واللوجستية، والتكنولوجية، والمتعلقة بكل من البيانات، والاتصالات. قاد فرق توزيع اللقاحات المحلية منسقون قاموا بإدارة التسجيل، والخدمات اللوجستية، والاتصالات، وإدارة اللقاحات. واستخدم البرنامج لقاحات كوفيد 19 المعتمدة لدى منظمة الصحة العالمية، ووضع معاييرًا لتحديد أولويات توزيع إمدادات اللقاح. وقد ضمن البرنامج عدم تحويل اللقاحات المخصصة لموظفي الأمم المتحدة عن الفئات الذين كان من المقرر تخصيصها لهم.

**النتائج:**

نجح البرنامج في تشكيل 120 فريقًا للتوزيع في 152 دولة وإقليمًا ومنطقة إدارية مؤهلة، مستهدفًا حوالي 673000 فرد. وبحلول أبريل/نيسان 2023، كان 72 دولةً وإقليمًا ومنطقة إدارية قد استقبلت أكثر من 470000 جرعة، تم حقن 337072 جرعة منها. وكان ما يقرب من نصف الجرعات المعطاة (167616) لأفراد في خمسة من دول الأمم المتحدة التي تعاني من الصعوبات. تم الإبلاغ عن خمسة وتسعين من حالات الآثار الجانبية الشديدة، لكن لم يؤد أي منها إلى الإنقاذ الطبي، أو العجز الدائم، أو الوفاة.

**الاستنتاج:**

أثبت البرنامج فعالية التنسيق العالمي والتنفيذ المحلي، والتكيف مع الأوضاع المتنوعة والتحديات التشغيلية. ويمكن أن يكون النموذج بمثابة دليل للأطراف الفاعلة العالمية لحالات الطوارئ الصحية المستقبلية، أو لتوزيع المساعدات الصحية على نطاق إقليمي أو عالمي.

## 摘要

**目的:**

介绍联合国（UN）的新型冠状病毒肺炎 (COVID-19) 疫苗接种计划，以及其为新型冠状病毒肺炎疫苗获取受限或无法获取的地区的一线人道主义工作者接种疫苗所做的努力。

**方法:**

疫苗接种计划分为两级运作：全球疫苗部署支持小组和在每个参与国家、领域或行政区域的当地疫苗部署小组。核心小组由一名全球疫苗协调员领导，负责监督医疗、法律、财务、后勤、数据、技术和通信方面的工作。当地疫苗部署小组由负责登记、后勤、通讯和疫苗管理工作的协调员领导。该计划使用了世界卫生组织批准的新型冠状病毒肺炎疫苗，并制定了分配供应疫苗的优先次序标准。该计划确保了所有联合国工作人员的疫苗不会从他们原计划进行注射的人群中被挪用。

**结果:**

该计划在 152 个符合条件的国家、领域和行政区域成功组建了 120 个部署小组，其目标为覆盖约 67.3 万人。截至 2023 年 4 月，已有 72 个国家、领域和行政区域收到超过 47 万剂疫苗，其中已完成接种 33,7072 剂。几乎一半的剂量（16,7616 剂）是用于联合国中五个贫困艰苦国家的个人接种。据报告发生了 95 起严重不良事件，但没有收到任何关于需要医疗后送、永久性残疾或死亡的报告。

**结论:**

该计划在适应不同的环境条件和应对操作挑战方面，表现出了有效的全球协调性和地方执行力。该模型可作为全球行动者在应对未来突发医疗事件或在区域或全球范围内部署医疗援助时的指南。

## Резюме

**Цель:**

Описать программу вакцинации против коронавирусной болезни 2019 года (COVID-19) Организации Объединенных Наций (ООН) и ее усилия по вакцинации гуманитарного персонала, находящегося на переднем крае работ, в местах с ограниченным или отсутствующим доступом к вакцине против COVID-19.

**Методы:**

Программа вакцинации была организована как двухуровневая операция: глобальная группа поддержки распределения вакцины и местные группы распределения вакцины в каждой участвующей стране, территории или административном районе. Главная группа, возглавляемая глобальным координатором по вакцинам, контролировала медицинские, юридические, финансовые, логистические, информационные, технологические и коммуникационные аспекты. Местные команды по распределению вакцин возглавляли координаторы, которые курировали вопросы регистрации, логистики, коммуникации и введения вакцин. В рамках программы использовались одобренные Всемирной организацией здравоохранения вакцины против COVID-19 и были разработаны критерии приоритетности при распределении вакцин. В рамках программы гарантировалось, что вакцины для персонала ООН не будут перенаправлены от населения, которому они предназначались.

**Результаты:**

В рамках программы было успешно сформировано 120 групп распределения в 152 отвечающих требованиям странах, территориях и административных районах с охватом около 673 000 человек. К апрелю 2023 года 72 страны, территории и административных района получили более 470 000 доз, из которых 337 072 дозы были введены. Почти половину доз (167 616) получили жители пяти стран, находящихся в трудном положении по версии ООН. Сообщалось о 95 серьезных нежелательных явлениях, но ни одно из них не привело к эвакуации по причинам медицинского характера, постоянной нетрудоспособности или летальному исходу.

**Вывод:**

В рамках программы была продемонстрирована эффективная глобальная координация и локальная реализация, адаптированная к различным условиям и эксплуатационным задачам. Модель может послужить руководством для глобальных игроков в случае возникновения чрезвычайных ситуаций санитарно-эпидемиологического характера в будущем или для развертывания медицинской помощи в региональном или глобальном масштабе.

## Introduction

The humanitarian workforce has been at the forefront of many, often concurrent crises, exposed to health and safety risks arising from their operations. These operations serve beneficiaries facing conflict, economic, social and climate crises, and recently the coronavirus disease 2019 (COVID-19) pandemic.^1^ To enable workers to stay and deliver on their mandates, humanitarian organizations have a duty to protect personnel safety, health and well-being, by implementing all necessary protection and control measures.^2^^–^^4^ Among protection measures against biological risks, vaccination has long been a recognized cost-effective prevention tool,^5^ typically administered before deployment to field missions, or at the occasion of local vaccination campaigns, such as against seasonal influenza. However, at the initial rollout of COVID-19 vaccines, organizations had limited access to vaccines where the humanitarian workers were operating due to the unequal global vaccine distribution.^6^^,^^7^

To address this challenge, the United Nations (UN) created the UN System-wide COVID-19 Vaccination Programme as part of its overall COVID-19 duty of care response.^8^^–^^10^ The aim of the programme was to procure, deliver and administer vaccines where access to vaccines was limited or completely absent to UN humanitarian workers, peacekeepers, retired staff as well as individuals working for associated nongovernmental implementation partners.^11^ The programme was intended to enable humanitarian personnel to stay and deliver on critical mandates, and to contribute to ongoing recovery work from the pandemic. Here we describe the programme, the lessons learnt and success factors for application to future pandemics.

## Methods

### Vaccination programme design 

The UN developed the vaccination programme as a two-level operation to best support its global scope of work. The programme’s published framework describes the overall programme set-up.^12^

At the central level, a cross-functional and inter-entity UN working group, known as the global vaccine deployment support team, was established. The group, which met through virtual meetings, was led by a global vaccine coordinator overseeing all functions of the programme, including medical, legal, financial, logistical, data, technological and communication aspects. The central support team contracted a global freight forwarding company, based in Denmark, to coordinate all vaccine shipments. 

At the local level, each participating UN country team was invited by the central support team to set up a local vaccine deployment team led by a coordinator overseeing all local functions. The UN country teams appointed local vaccine deployment coordinators from different participating UN entities, based on the coordinators’ respective expertise and availability.^13^ The coordinators collaborated with in-country stakeholders to guarantee the registration of eligible individuals and to ensure the receipt, handling, transportation and storage of vaccines. They also made sure that vaccine administration arrangements and communication plans were established in the country.

At both the central and local levels, emphasis was laid on working together as one unified organization, by breaking down silos across the UN entities and leveraging various expertise and resources. Overall, 32 UN entities^14^ participated in the programme, with 31 of them entering a memorandum of understanding with the UN Secretariat. A central cost-share mechanism secured funding for the programme, thus allowing the country teams to participate with little or no impact on local budgets for their eligible populations.

Recognizing variations in local factors between countries such as the type and number of UN facilities (especially medical facilities), population size and location, the central support team developed different implementation models. In countries, territories or administrative areas with a small population served by only one UN facility, the vaccine deployment team consisted of four individuals who addressed all the components of the programme: planning, communication, pre-registration, administration, transportation and storage of the vaccine. In countries, territories or administrative areas with medium-to-large populations, often with multiple duty stations and/or served by several UN entities, communication and registration activities were centralized in one location. Meanwhile focal points from different duty stations, appointed by local vaccine deployment teams, managed the vaccination site(s), administration of vaccines, and handling and maintenance of vaccine stocks. In countries, territories and administrative areas with no UN health-care facility, the local team had to identify a medical facility and/or contractor to administer the vaccine.

The programme used COVID-19 vaccines approved by the World Health Organization (WHO) Emergency Use Listing Procedure.^15^

### Vaccination strategy

Due to the limited quantity of vaccines initially made available to the programme, the central support team in conjunction with the UN Medical Directors group developed criteria to prioritize the countries, territories and administrative areas and individuals within them who would be vaccinated first. This prioritization was done using an occupational health and operational risk-based approach aligned with the *World Health Organization (WHO) Strategic Advisory Group of Experts on Immunization (SAGE) roadmap for prioritizing uses of COVID-19 vaccines in the context of limited supply*.^16^

First, to prioritize the countries, territories and administrative areas, the working group developed a prioritization model^17^ assessing countries, territories and administrative areas across six weighted health and safety parameters (Table 1). Five parameters were internal to the UN system: (i) level of access of humanitarian personnel to local UN or other health-care services; (ii) security level as defined by internal security services; (iii) COVID-19 medical evacuation rate; (iv) country mobility and hardship classification; and (v) COVID-19 case reporting rates. The external parameter was the human development index.^19^ Economic classifications of countries, territories and administrative areas were not included in the prioritization. Selection of the parameters was based upon a published literature review^20^ that revealed the key factors for a country’s lack of vaccine access, which included inadequate local public health and medical infrastructure or the collapse thereof; inadequate training of local health-care workers; and levels of conflict or violence. The group also consulted an article describing how these parameters affect a vaccine campaign. ^21^ The central support team calculated the individual country scores through the addition of all weighted indicator metrics. The initial list contained 50 top priority countries, territories and administrative areas. After assessing the operational capacities to receive, store and administer vaccines, the central support team adapted the list to include only countries, territories and administrative areas with such capacity.

**Table 1:** Criteria used to prioritize countries, territories and administrative areas to receive COVID-19 vaccines from the UN COVID-19 vaccination programme

Criteria	Interpretation	Range	Weight %
**Internal to the UN system**			
First line of defence index	Level of access for local UN personnel to the local UN clinics and/or other health-care services based on qualitative assessments performed by UN country teams at the beginning of the pandemic^18^	1 (low)–5 (high)	30
Security level system	Indicator grading level of threat in the areas of armed conflict, terrorism, crime, civil unrest or human-made natural hazards	1: minimal 2: low 3: moderate 4: substantial 5: high 6: extreme	25
Medical evacuation rate	Rate-based indicator calculated from the number of COVID-19 medical evacuations among UN personnel, dependents and international NGO personnel, divided by the total population in that country	Rate-based	20
Mobility hardship	Difficulty of working and living conditions at duty station	A (low difficulty) – E (high difficulty)	10
COVID-19 case rate	Rate-based indicator calculated from the number of COVID-19 cases among UN personnel and dependents divided by the total population in that country	Rate-based	10
**External to the UN system**			
Human development index^19^	Measure of average achievement in the dimension of health, education and standard of living. Human development index is the geometric mean of normalized indices for each of the three dimensions	0 (natural zero) – 1 (aspirational target)	5

COVID-19: coronavirus disease 2019; NGO: nongovernmental organization; UN: United Nations.

Second, to determine which humanitarian workers’ groups should receive the vaccines, regardless of their duty station, the UN Medical Directors group developed an occupational health risk matrix, considering the exposure doses and frequency of different work categories. Occupational groups were classified as: high risk (high exposure dose and frequency); low risk (low exposure dose and frequency); and medium risk, comprising the remaining workers’ groups (Table 2).^22^ Assessment of workers’ health risks, such as age and health conditions,^16^ within each group was also considered.

**Table 2:** Risk of COVID-19 through occupational exposure by humanitarian workers' activities

Description	SAGE priority category^16^	Exposure dose level	Exposure frequency level	Overall risk	Examples of roles
**Contact with people or fluids with known, suspected or possible COVID-19**					
Medical, post-mortem, or laboratory-related activities	Ia	High	High	High	Most health workers caring for those with respiratory illness
Work with COVID-19 patients in crowded, enclosed places without adequate ventilation or where aerosol generating procedures are performed	Ia	High to very high	High to very high	High	Clinic or hospital doctor, surgeon, nurse, other paramedical and support personnel
Physical examination and providing direct care for a known or suspected COVID-19 patients	Ia	High	High	High	Doctor, nurse, physician assistant
Testing services using manipulated respiratory samples	Ia	High	High	High	Sample testing staff, laboratory technician
Handling stool, urine or waste, or cleaning equipment associated with COVID-19 patients	Ia	High	High	High	Clinic or hospital nursing, laboratory, technical, cleaning, laundry and similar support staff
Transportation of patients known or suspected to have COVID-19 without adequate distancing	Ia	High	High	High	Ambulance officers or drivers, aviation officers conducting medical evacuation^a^
Physical examination of or face-to-face contact with patients without symptoms suggestive of COVID-19	Ia	Low	High to very high	High	Clinic doctor, nurse, patient-facing receptionist
Clinical staff providing vaccinations	II	Low	High	High	Recognizes ill people may seek vaccination and downplay symptoms
**Non-medical roles**					
Frequent contact with people with unknown status	III	Varies	Varies	Medium	NA
Frequent close or relatively uncontrolled interaction with communities in crowded settings with limited physical distancing	III	Low	High	Medium	Workers with field activities such as refugee registration; field monitoring; food voucher and cash distribution; and education
Frequent and close interaction with general public or co-workers	III	Low	High	Medium	Security officer, maintenance, drivers, cafeteria workers
Those required to live in close quarters	III	Low	High	Medium	Troops
Emergency response	III	High	Low	Medium	Security officer, other first responders
Non-public-facing activities with infrequent contact with people with unknown status	Not Classified	Low	Low	Low	NA
Can work remotely and live at home or has only occasional controlled contact with public	Not Classified	Low	Low	Low	Administrative staff, other back-office staff

COVID-19: coronavirus disease 2019; NA: not applicable; SAGE: Strategic Advisory Group of Experts on Immunization.

^a^ Medical evacuation is the urgent transportation of an ill individual to another country when essential care or treatment cannot be provided locally due to inadequate medical facilities.

Note: SAGE priorities categories are: Ia: Stage Ia includes health workers at high or very high risk of acquiring and transmitting infection; II: Stage II includes health workers engaged in immunization delivery; and III: Stage III includes social or employment groups at elevated risk of acquiring and transmitting infection.^16^

### Target population

The central support team identified countries that had UN personnel stationed in their country and had absent or low access to vaccine (Box 1). In these countries, all personnel of the 32 participating UN entities and their dependents; UN system retirees; military and police personnel deployed by the UN; key contractors; as well as nongovernmental organizations (NGOs) and other implementing partners sponsored by participating UN entities were eligible.^24^ To estimate the number of individuals eligible it was necessary to assess the size of UN entities operating in each country. The primary data source was records from internal security services, which had aggregate numbers of UN personnel and dependents in each country. Subsequently, as more partner organizations became eligible for participation in the programme through UN sponsorship and initially unrecorded dependents were identified, the estimated number increased.

Box 1 **Countries, territories and administrative areas included in the UN system-wide COVID-19 vaccination programme, by participation and income group, 2021**

**Initially targeted, but did not participate^a^**

Low income: Burkina Faso; Chad; Democratic People's Republic of Korea; Gambia; Liberia; Rwanda; Sierra Leone; Togo; Uganda; and Zambia.

Lower-middle income: Algeria; Angola; Benin; Bhutan; Cambodia; Congo; Côte d'Ivoire; Egypt; Eswatini; Ghana; India; Indonesia; Kiribati; Micronesia (Federated States of); Mongolia; Morocco; Pakistan; Senegal; Solomon Islands; Vanuatu; and West Bank and Gaza Strip.

Upper-middle income: Azerbaijan; Belize; Botswana; China; Dominica; Dominican Republic; Equatorial Guinea; Fiji; Georgia; Grenada; Guyana; Iraq; Jordan; Kazakhstan; Malaysia; Maldives; Marshall Islands; Mauritius; Mexico; Montenegro; Namibia; Palau; North Macedonia; Russian Federation; Saint Lucia; Saint Vincent and the Grenadines; Serbia; South Africa; Suriname; Tonga; Türkiye; Turkmenistan; and Tuvalu.

High income: Antigua and Barbuda; Aruba; Bahamas; Bahrain; Barbados; Chile; Kuwait; Nauru; Panama; Saint Kitts and Nevis; Saudi Arabia; Seychelles; Singapore; United Arab Emirates; and Uruguay.

Uncategorized: Cook Islands and Venezuela (Bolivarian Republic of).

**Participating in Phase I**

Low income: Madagascar and Yemen.

Lower-middle income: Bolivia (Plurinational State of); Cabo Verde; Comoros; Djibouti; El Salvador; Haiti; Honduras; Kyrgyzstan; Lao People's Democratic Republic; Lebanon; Mauritania; Nigeria; Sri Lanka; Tajikistan; Ukraine; Uzbekistan; and Viet Nam.

Upper-middle income: Albania; Argentina; Armenia; Bosnia and Herzegovina; Brazil; Colombia; Ecuador; Gabon; Jamaica; Kosovo; Republic of Moldova; Paraguay; Peru; and Thailand.

High income: Trinidad and Tobago.

**Participating in Phase II**

Low income: Eritrea and Malawi.

Lower-middle income: Bangladesh; Cameroon; Lesotho; and Samoa.

**Participating in Phase I and II**

Low income: Abyei Area Administration, Afghanistan; Burundi; Central African Republic; Democratic Republic of the Congo; Ethiopia; Guinea; Guinea-Bissau; Mali; Mozambique; Niger; Somalia; South Sudan; Sudan; and Syrian Arab Republic.

Lower-middle income: Iran (Islamic Republic of); Kenya; Myanmar; Nepal; Nicaragua; Papua New Guinea; Philippines; São Tomé and Príncipe; United Republic of Tanzania; Timor-Leste; Tunisia; and Zimbabwe.

Upper-middle income: Belarus; Costa Rica; Cuba; Guatemala; and Libya.

COVID-19: coronavirus disease 2019, UN: United Nations.^a^ The programme did not document reasons for not participating, but most country teams expressed that the vaccine was available through other sources.Note: We used the 2021 World Bank Group classification^23^ to categorize country by income level. Countries, territories and administrative areas in the uncategorized group had no reported classification.

### Data collection and recording

To identify and register eligible individuals, the central support team developed specifications for creating a cloud-based electronic record system. The programme outsourced the work of building the platform to a third-party provider. The platform included functionalities to collect and record consent forms; record risk categories from the occupational health risk matrix, as well as age and health risk factors; schedule appointments; record vaccination administration; document adverse events; and issue electronic vaccination certificates in a format consistent with WHO standards.^25^ The platform and the electronic certificates were in compliance with Personal Data Protection and Privacy Principles.^26^

During the two years the programme was active, UN information technology personnel, part of the central support team, were responsible for adapting the platform to meet the needs of the programme throughout all its phases and to meet needs of the platform users to improve user-experience. These changes included revisions to questions such as marking them as mandatory or voluntary to submit responses to; incorporating the consent and release from liability forms into the registration rather than having them signed separately; expanding self-registration eligibility; and making all platform data visible to local coordinators.

### Communication plan

The central support team created a publicly accessible website,^11^ which was regularly updated to provide all relevant information, resources and guidance to personnel and local country teams. The vaccination schedule was published in advance and updated regularly on the website.^11^ The programme also published a document with frequently asked questions in multiple languages.^27^ Video interviews with UN officials and medical personnel, relayed by the network of vaccination teams, provided further information regarding the vaccination programme and its registration process, available vaccines, vaccine administration and vaccine certificates. 

At every duty station, a communication focal point was responsible for the execution of the programme’s communication strategy. The focal point provided local personnel and officials with a set of clear messages developed by the central support team, through emails or town hall sessions. The messages included eligibility criteria; vaccine specifics (vaccine name, type, WHO Emergency Use Listing Procedure approval); vaccine arrival timeline; and the purpose of the vaccination. The focal points also encouraged vaccination while emphasizing its voluntary nature. To ensure alignment with national programme schedules, the focal point worked closely with country health officials. The programme also organized communication events for the UN populations, where the attendants received information about programme updates, vaccine availability and any additional timely information that would raise awareness, and answer questions. 

### Ethical considerations

Implementation of the vaccination programme for the UN population targeted areas where supply was expected to be limited or absent. This setting led to ethical considerations on how to best protect frontline workers and partners while ensuring equitable access to the vaccine by local populations. In addition to following the WHO SAGE Roadmap,^16^ the decision-makers in each location were carefully considering the progress of the national vaccine rollouts, the timing of vaccine deliveries^27^ and the relevance of complementary national vaccination efforts when deciding to deploy vaccines, and the timing of administration. Communication messages directed primarily to UN personnel regularly emphasized that the UN personnel were expected to follow national or UN-related prioritization guidelines; that keeping the UN personnel healthy and safe reduced strain on local health infrastructures and allowed them to focus on implementing their mandates to serve and protect others; and that vaccines for the UN personnel were not diverted from the populations they were intended to serve, as the supply was obtained independently of national or COVID-19 Vaccines Global Access (COVAX) facility allocations.

### Data availability

Data can be made available upon request, subject to consideration for maintaining the anonymity of participants.

## Results

**Fig. 1:**
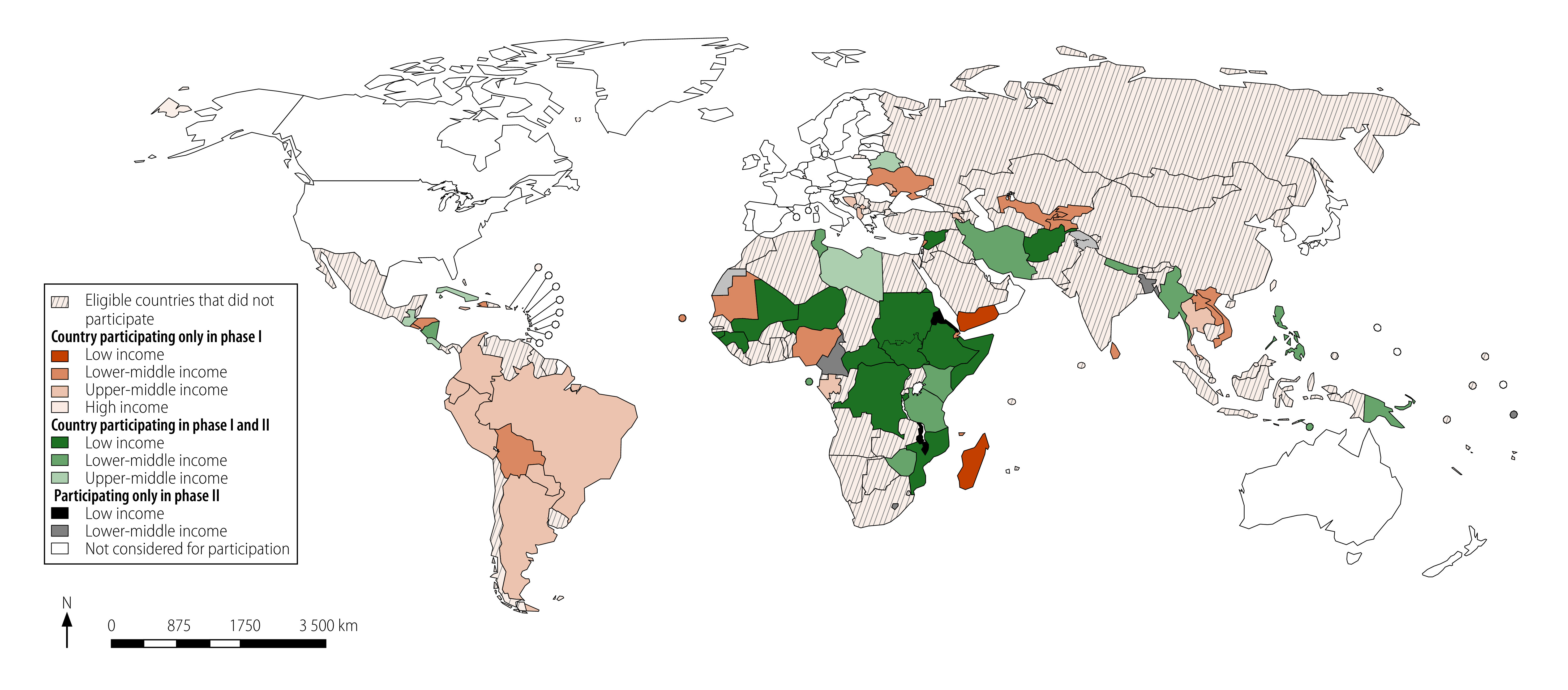
Map of eligible countries for UN COVID-19 vaccination programme

COVID-19: coronavirus disease 2019; UN: United Nations.

Note: We used the 2021 World Bank Group classification^23^ to categorize countries, territories and administrative areas by income level. In 2021, there was no World Bank Group classification by income groups reported for the Cook Islands and Bolivarian Republic of Venezuela. Box 1 presents the countries, territories and administrative areas included in the UN COVID-19 vaccination programme.

In total, the programme formed 120 deployment teams tasked with vaccinating a target population of approximately 673 000 individuals across 152 countries, territories and administrative areas, including 503 255 UN individuals and 159 719 personnel from partner organizations (Fig. 1). Out of them, 20.0% (134 400 individuals) met the high-risk prioritization criteria.

In early March 2021, the programme acquired 300 000 doses of the Covishield™ vaccine (AstraZeneca, Cambridge, United Kingdom of Great Britain and Northern Ireland) manufactured by the Serum Institute of India (Pune India), from two different lots with relatively short shelf lives (20 July and 23 August 2021). Subsequently, the programme acquired the vaccines Jcovden (Janssen, Beerse, Belgium); BBIBP-CorV (Sinopharm, Shanghai, China); and Spikevax (Moderna, Cambridge, United States of America).

### Administration and coverage

In the first phase, which occurred between 1 April and 31 August 2021 when COVID-19 vaccines were not readily available, 300 000 doses of the Covishield™ vaccine were delivered in over 100 shipments to 66 countries, territories and administrative areas that met the prioritization criteria. Approximately 250 000 doses of the administered doses were recorded in the cloud-based platform, while another 15 000 administered doses were manually recorded during vaccination, and later the central support team transferred these records to the platform. 

The programme redistributed 38 510 excess doses from countries, territories and administrative areas with surplus vaccines to others in need between July and August 2021. Another 23 000 doses were reported to the central support team as donated or swapped for doses from other sources with longer shelf lives.

From September 2021 and onwards, the programme entered phase II when it evolved from a model seeking to provide vaccines to as many local teams as possible for primary immunization, into a model where local teams could request additional doses for any residual primary series, booster doses or variant-containing vaccines.^28^ As national programmes became more robust, several local teams active in phase I suspended their participation and directed their personnel to alternate options available in their respective countries, territories and administrative areas. In the second phase, up to 1 April 2023, another 172 661 doses of different vaccine types were delivered across 38 countries, territories and administrative areas.

As of 1 April 2023, the programme has delivered over 470 000 vaccine doses to 72 countries, territories and administrative areas (Fig. 1) and 337 072 doses were recorded as administered. Out of these, 193 871 individuals have received at least one dose while 121 063 have received at least two doses of COVID-19 vaccine through the programme (Table 3).

**Table 3:** Cumulative number of individuals who have received COVID-19 vaccine through the UN system-wide COVID-19 Vaccination Programme, 1 April 2023

Eligible group, by country income classification	No. of people vaccinated						Total no. of doses administered
	One dose	Two doses	Three doses	Four doses	Five doses	Total	
**Low-income countries, territories and administrative areas**							
Civilian personnel	14 296	16 245	4 628	438	2	35 609	62 432
Uniformed personnel	9 742	32 946	6 677	184	0	49 549	96 401
International NGO partners	2 513	2 380	108	1	0	5 002	7 601
Dependents of UN personnel	1 297	1 986	165	21	0	3 469	5 848
Dependents of international NGO partners	204	164	7	1	0	376	557
Retirees	132	94	8	0	0	234	344
Sub-total	28 184	53 815	11 593	645	2	94 239	173 183
**Lower-middle income countries and territories **							
Civilian personnel	11 261	9 530	2 045	29	0	22 865	36 572
Uniformed personnel	250	3 700	0	0	0	3 950	7 650
International NGO partners	10 866	5 480	3 498	1	0	19 845	32 324
Dependents of UN personnel	5 861	3 446	855	6	0	10 168	15 342
Dependents of international NGO partners	2 546	401	103	1	0	3 051	3 661
Retirees	202	229	45	0	0	476	795
Sub-total	30 986	22 786	6 546	37	0	60 355	96 344
**Upper-middle-income countries and territories**							
Civilian personnel	2 423	8 225	886	44	0	11 578	21 707
Uniformed personnel	265	194	91	1	0	551	930
International NGO partners	236	698	47	0	0	981	1 773
Dependents of UN personnel	833	1 987	276	14	0	3 110	5 691
Dependents of international NGO partners	147	188	15	0	0	350	568
Retirees	73	50	19	2	0	144	238
Sub-total	3 977	11 342	1 334	61	0	16 714	30 907
**High-income countries and territories**							
Civilian personnel	11	128	0	0	0	139	267
Uniformed personnel	0	0	0	0	0	0	0
International NGO partners	0	0	0	0	0	0	0
Dependents of UN personnel	0	0	0	0	0	0	0
Dependents of international NGO partners	0	0	0	0	0	0	0
Retirees	0	0	0	0	0	0	0
Sub-total	11	128	0	0	0	139	267
**Uncategorized^a^**							
Civilian personnel	3 071	3 913	537	30	0	7 551	12 628
Uniformed personnel	5 249	6 429	458	22	0	12 158	19 569
International NGO partners	460	306	6	1	0	773	1 094
Dependents of UN personnel	747	816	49	1	0	1 613	2 530
Dependents of international NGO partners	53	80	3	0	0	136	222
Retirees	70	114	6	3	0	193	328
Sub-total	9 650	11 658	1 059	57	0	22 424	36 371
**Programme total**	**72 808**	**99 729**	**20 532**	**800**	**2**	**193 871**	**337 072**

COVID-19: coronavirus disease 2019; NGO: nongovernmental organization; UN: United Nations.

^a^ Individuals moving location and/or economic classification was not applicable to location.

Note: Countries, territories and administrative areas were classified according to the World Bank economic classification.^23^

The UN vaccination teams in Myanmar administered the largest number of doses through the programme (40 081 doses to 21 696 persons; Table 4). More than 67.1% (26 907) of these doses were administered to implementing partners across 260 NGOs.

**Table 4:** Cumulative doses administered under the UN COVID-19 vaccination programme, Myanmar, as of 1 April 2023

Eligible group	No. of doses administered (%)	Percent of all doses delivered in country (*n* = 65 170)	Total no. of individuals vaccinated
International NGO partners	26 907 (67.1)	41.3	15 454
Civilian personnel	8 226 (20.5)	12.6	3 854
Dependents of UN personnel	4 206 (10.5)	6.5	2 042
Dependents of international NGO partners	524 (1.3)	0.8	246
Retirees	218 (0.5)	0.3	100
**Total**	**40 081 (100.0)**	**61.5**	**21 696**

COVID-19: coronavirus disease 2019; NGO: nongovernmental organization; UN: United Nations.

Note: Inconsistencies arise in some numbers due to rounding.

Other hardship countries with high quantities of vaccines administered were, in descending order, South Sudan, Mali, Central African Republic and Democratic Republic of the Congo. In these countries, the programme vaccinated 42.2% (69 725/165 399) of the eligible population across the two-year period. At least one dose of vaccine was administered to 47 454 high-risk individuals in these four countries, which accounts for 35.3% of the 134 400 people assigned as high-risk population in the programme (Table 5).

**Table 5:** Low-income hardship countries with the greatest number of delivered and administered doses from the UN COVID-19 vaccination programme, as of 1 April 2023

Country	Prioritization rank^a^	No. of doses delivered^b^	No. of doses administered^b^ (% of doses administered globally)^c^	Eligible population at the start of phase I,^d^ no.	Vaccinated eligible population,^b^ no. (%)^e^	High-risk individuals vaccinated during phase I^f^
Central African Republic	1	37 990	31 672 (9.4)	26 750	17 510 (65.5)	11 030
South Sudan	4	59 810	39 551 (11.7)	58 535	20 502 (35.0)	16 606
Mali	8	37 770	31 448 (9.3)	30 082	16 430 (54.6)	12 006
Democratic Republic of the Congo	28	26 240	24 864 (7.4)	50 032	15 283 (30.5)	7 812
**Total**	**NA**	**161 810**	**127 535 (37.8)**	**165 399**	**69 725 (42.2)**	**47 454**

COVID-19: coronavirus disease 2019; NA: not applicable; UN: United Nations.

^a^ Prioritization ranking of countries is based on criteria presented in Table 1.

^b^ Includes phase I and II.

^c^ The UN COVID-19 vaccination programme administered a total of 337 072 doses as of 1 April 2023.

^d^ Eligible population size was estimated during the programme’s set up and was not updated further to reflect any changes in personnel size.

^e^ The percentages show the total vaccinated population over phase I and II to the eligible population reported at the start of phase I. The actual vaccinated population may be greater as the cloud-based platform only recorded doses administered through the programme and did not track individuals if they received doses from national programmes.

^f^ Vaccinated with at least one dose.

### Costs

The programme relied on resources donated by the participating UN organizations. For example, several appointed country focal points were medical professionals already employed by the UN entities, and logistics, procurement, or information technology functions were supported by existing capacities. Notably, some vaccine doses were also donated. 

The programme had to seek funding of about 6 million United States dollars to cover the costs of other key elements such as additional vaccine doses; ancillary items (e.g. syringes, needles); third-party services for temperature-controlled vaccine storage and shipment; the cloud-based platform and salaries for a few dedicated personnel who helped running the programme. 

### Adverse events 

Of the 337 072 doses administered, 12 289 identified adverse events were reported in the platform. The top three reported non-serious events were fever (3146; 25.6%), headache (2133; 17.4%) and general body aches or pain (1774; 14.4%).

A total of 95 (0.8%) serious adverse events were reported that either required urgent medical attention such as hospitalization, or were potentially life-threatening or resulted in a disability.^29^ Among these, there were 24 instances of hospitalization (0.2%); and 51 instances of cardiac-related issues (0.4%) such as chest tightness (7; < 0.1%), heart palpitations (12; 0.1%) and chest pain (16; 0.1%). Severe adverse events were rare and, to the best of the central support team’s knowledge, did not lead to any medical evacuation, permanent disability or death.

## Discussion

Although the UN System-wide COVID-19 Vaccination Programme was able to procure a substantial number of doses, it was not enough to fully vaccinate the total eligible population. In the first phase, the programme focused on vaccinating the individuals identified as high-risk population, of which about one third of them were from four hardship countries. In the end, the programme was able to successfully administer about half of its allocated doses within hardship countries, and also extend access to NGO partners. 

As the programme procured additional doses and vaccines became increasingly available through national vaccination efforts, other less-priority groups were able to access vaccination. The programme used internal UN parameters such as rate of medical evacuations, security and hardship classification to prioritize countries, territories and administrative areas. While this prioritization can be difficult to replicate in other contexts, our occupational health risk matrix developed to prioritize occupational groups could be applied to other occupational settings.

The seemingly low proportion of individuals vaccinated (193 871 out 673 000 eligible individuals) is an underrepresentation of the actual vaccination coverage of the eligible population for several reasons. The reporting platform only recorded doses administered through the programme and did not account for vaccinations received by eligible individuals through other efforts, to which the humanitarian workforce progressively gained more access during the study period. Vaccine hesitancy also likely played a role during the initial phase where the programme’s only available vaccine was the Covishield™ vaccine. Reports of the rare but severe vaccine-induced thrombotic thrombocytopenia in healthy young individuals,^30^ and reduced vaccine efficacy,^31^ led several African countries to go against WHO recommendation and ban or restrict the vaccine for younger age groups, many of whom were frontline workers. The programme addressed vaccine hesitancy through a combination of vigorous internal global communication and local campaigns run by the UN country teams, highlighting vaccination benefits over risks of side-effects. The involvement of the UN country teams and their coordinators (through role modelling, organization of town hall meetings and emails to participants), the participation of WHO experts, and the systematic use of WHO and other official written guidance was especially beneficial.^32^^–^^34^

The programme faced logistical challenges such as short expiry dates limiting the timeline for second doses; customs clearance delays; cold chain and storage requirements; and limited internet connectivity in hardship countries, territories and administrative areas. Strategies to address these challenges included close coordination between the local vaccination teams, national authorities and senior in-country UN officials; leveraging the expertise of each UN entity; swift reallocation of surplus unused doses to other locations for administration before their expiration dates; and optimization of vaccine administration by using the latest WHO guidance on mix-and-match schedules that allowed individuals to complete primary vaccination schedules with a different WHO Emergency Use Listing Procedure vaccine found outside the programme, or a vaccine with a longer expiry date.^35^ Furthermore, the accurate tracking of the number of high-risk individuals remained challenging throughout the programme due to personnel movements, and updates to the cloud-based platform that simplified the registration as the eligibility criteria broadened in the second phase. In the future, vaccination programmes should tailor the reporting platforms to ensure consistent recording of risk-prioritization, even as the eligibility might expand.

The wastage of vaccine was reasonable, estimated at no more than 5% in the first phase, and well within forecast rates of 10% and the wastage rates of up to 30% reported by other vaccination campaigns.^36^ As for the second phase, the wastage rate is yet to be determined, as the programme continues to hold an inventory of unexpired vaccines.

The UN and other organizational entities regularly conduct local vaccination drives for their personnel, but also roll out exceptional distribution of supplies or vaccinations during outbreaks, such as personal protective equipment during Ebola virus disease outbreaks and cholera vaccines. Based on our experiences, a model for large-scale operations can be developed to efficiently manage emergency health aid and immunization campaigns across multiple countries, territories and administrative areas. This model would integrate routine and endemic disease vaccines, particularly in regions where local procurement is challenging.^37^

In conclusion, the programme has been contributing to protecting the frontline humanitarian workforce and peacekeepers in the context of an exceptional global health emergency, and enabling them to continue delivering on their critical mandates to protect, serve and save others. The model was quickly developed with no pre-established protocol and enabled rapid distribution and administration of vaccines across the world, especially in hardship countries, territories and administrative areas. The programme’s success can be attributed to its two-level operational structure, rigorous risk-based prioritization approach, flexible logistic and vaccine administration model, collaborative use of in-house experts and close coordination across various UN entities. The programme provides an example of working together as One UN across entities on both a global and local scale.
